# Structure and Mechanical Properties of a Porous Polymer Material via Molecular Dynamics Simulations

**DOI:** 10.3390/polym15020358

**Published:** 2023-01-10

**Authors:** Sharon Carol Volpe, Dino Leporini, Francesco Puosi

**Affiliations:** 1 Dipartimento di Fisica ‘Enrico Fermi’, Università di Pisa, Largo B. Pontecorvo 3, 56127 Pisa, Italy; 2 Istituto per i Processi Chimico-Fisici-Consiglio Nazionale delle Ricerche (IPCF-CNR), Via G Moruzzi 1, 56124 Pisa, Italy; 3 Istituto Nazionale di Fisica Nucleare, Largo B. Pontecorvo 3, 56127 Pisa, Italy

**Keywords:** porous materials, phase separation, glass transition, structural analysis, mechanical properties, molecular dynamics

## Abstract

We characterize, using molecular dynamics simulations, the structure and mechanical response of a porous glassy system, obtained via arrested phase separation of a model polymer melt. In the absence of external driving, coarsening dynamics, with power-law time dependence, controls the slow structural evolution, in agreement with what was reported for other phase-separating systems. The mechanical response was investigated in athermal quasi-static conditions. In the elastic regime, low values for the Young’s and shear modulus were found, as compared to dense glassy systems, which originate from the porous structure. For large deformations, stress–strain curves show a highly intermittent behavior, with avalanches of plastic events. The stress-drop distribution is characterized exploring a large set of parameters. This work goes beyond the previous numerical studies on atomic porous materials, as it first examines the role of chain connectivity in the elastic and plastic responses of materials of this type.

## 1. Introduction

When a liquid is quenched inside the spinodal region, an off-equilibrium region of the phase diagram, phase separation occurs, decomposing the fluid into phases of different compositions. For the gas–liquid spinodal decomposition, the process can be interrupted via a deep virtually instantaneous quench in temperature. Indeed, if the target temperature is below the glass transition of the dense phase of the system [1], the dynamics of this phase are dramatically slowed down, and as it approaches the glassy state, phase separation is stopped. This leads to the creation of an amorphous porous bicontinuous structure, whose morphology depends on density and target temperature during the quench.

Albeit there is no fundamental predictive theory able to describe specific features of arrested spinodal decomposition, this mechanism is extensively used in experiments and simulations to produce distinctive morphologies [2,3,4,5,6]. Notably, extensive numerical work, based on molecular dynamics (MD) simulations, has been done by Testard et al. [7], aiming at the characterization of viscoelastic phase separation in a Lennard–Jones atomic binary fluid, to study the influence of the temperature quench on the liquid–gas phase separation kinetics in a Lennard–Jones fluid, and therefore the competition between the phase separation kinetics and the glass transition occurring at low temperatures in bulk liquids. Using a combination of direct visual inspection and proper quantitative methods to analyze the morphology of biphasic atomistic configurations, they determined the binodal and spinodal lines on the temperature-density phase diagram of the system. They also reported that the phase-separation kinetics change qualitatively with decreasing temperature, from surface-tension-driven diffusion for shallow quenches to spatially heterogeneous and thermally activated intermittent coarsening at low temperatures.

Building on this model, more recently, MD simulations have been carried out by Priezjev et al. [8,9] to investigate the mechanical behavior of a porous structure obtained by phase separation in the elastic and the plastic regimes. The stress–strain curves show linear behavior for small deformation, up to a yielding point, when the plastic regimes set in. The elastic modulus follows a power-law dependence on the average glass density, in agreement with theoretical predictions. It is also shown that upon tensile loading, breaking events occur in regions where lower glass density is present to a larger spatial extent.

The Lennard–Jones binary mixture is known to be a quite generic model, being able to reproduce several features of glassy phenomenology, regarding both the dynamics and the mechanical properties. Yet, the interesting question is to understand how molecular connectivity influences the phase-separation dynamics of the system and the subsequent mechanical response. In this work, we address this point using MD simulations to investigate the viscoelastic phase separation and mechanical properties of a coarse-grained model of polymer material. In addition to the small-deformation elastic regime, we explore in detail the onset and development of plasticity up to deformations as large as 50%.

## 2. Methods and Simulation

Molecular dynamics simulations were performed for a melt of linear polymer chains with M = 20 monomers each. The total number of monomers was kept fixed as N = 2 $\times \;10^{4}$. Non-bonded monomers, i.e., non-adjacent monomers in the same chain and monomers belonging to different chains, interact via a Lennard–Jones (LJ) potential:

(1) $$U_{LJ}\left(r\right)=4\epsilon \left[{\left(\frac{\sigma }{r}\right)}^{12}-{\left(\frac{\sigma }{r}\right)}^{6}\right]\;,\;r<r_{c}$$

where *r* is the distance between the monomers, σ the zero-crossing distance of the potential and $r_{c}=2.5\sigma$ is the cutoff radius beyond which the potential is set to zero for computational convenience. Bonds subsist between adjacent monomers in the same chain, which are modeled with a harmonic potential:

(2) $$U_{b}\left(r\right)=k{\left(r-r_{0}\right)}^{2},$$

with $k=555\epsilon /\sigma^{2}$ and $r_{0}=0.97\sigma$. As we focus on a fully flexible model, no torsional or bending potentials are included.

In the following, we used Lennard–Jones-reduced units, expressing lengths in units of σ; temperatures in units of $\epsilon /k_{B}$, with $k_{B}$, the Boltzmann constant and ϵ the energy scale of the LJ interaction; and time in units of $\tau_{MD}=\sqrt{m\sigma^{2}/\epsilon }$. We set $k_{B}=\sigma =\epsilon$ = m = 1.

Simulations were carried out using the open-source software LAMMPS [10]. We started from a homogeneous melt at fixed number density ρ=N/V. The system was initially equilibrated at high temperature T = 3.0 within the canonical ensemble (i.e., the NVT ensemble, with constant number of particles, volume and temperature), using the Nosé–Hoover thermostat [11,12]. Then, we performed an instantaneous (i.e., in a single time step) quench of the desired target temperature. The system was allowed to age up to $10^{6}$ time units within the NVT ensemble, with the temperature controlled using a Langevin thermostat [13]. Different values of the system density, ρ=N/V=0.3,0.4, and 0.6, and quench temperature, T=0.015,0.1, and 0.3, were considered, below the glass transition temperature of the model, which was estimated as $T_{g}\approx 0.4$ [1]. For each state point, 10 independent samples of the system were simulated to improve the statistics of the analysis.

Simulations for systems with M=5,10 were also performed to analyze the behavior of the system when approaching the atomistic liquid system. Here, we present the results for M=20, unless differently specified.

## 3. Results and discussion

### 3.1. Structural Analysis

**Figure 1:**
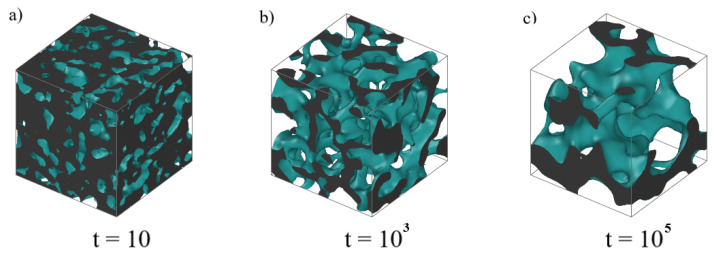
Morphologies obtained by letting the system age up to a time t (LJ units) after a quench at T = 0.1, ρ=0.6, (**a**) t = 10, (**b**) t = $10^{3}$, (**c**) t = $10^{5}$. To ease the visualization, a surface mesh was constructed using OVITO [14]. The green region represents the surface of the glassy phase. The gray region represents the inside of the dense phase sliced at the boundaries.

First, we analyze the morphologies resulting from the quench at different state points. Representative configurations are displayed in Figure 1. To ease the visualization, a surface mesh was constructed. The glassy domains appear to be a bicontinuous, highly interconnected network; gas domains are far from being spherical or regular in size. Typical length scales change with varying densities and temperatures. As the density increases, the glassy phase occupies an increasing volume fraction, and smaller length scales characterize the gas phase. The same effect can be observed for decreasing temperature, due to a more effective slowing down of dynamics, which prevents the progress of coarsening.

**Figure 2:**
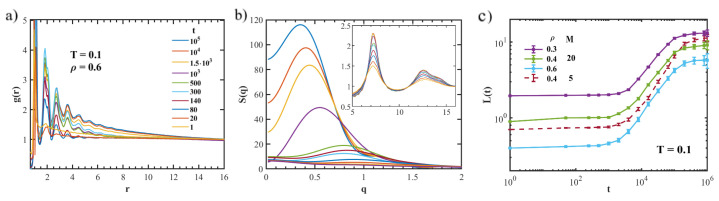
Panel (**a**): radial distribution function g(r), for a sample at T=0.1, ρ=0.6, at different aging times t. Panel (**b**): Corresponding static structure factor S(q). Panel (**c**): Time evolution of the average domain size L(t) for systems with different density quenched at T=0.1. For comparison, data for a system with M=5 are also shown (dashed line).

To characterize the structure of the system, we examine the pair correlation function g(r). In Figure 2a, we show the g(r) at different aging times for a sample with T=0.1 and ρ=0.6. In the porous system, the amorphous structure is denoted by an oscillating decay of pair correlations, with the amplitude of oscillations growing as the system ages. The position of the first crossing of the level g(r)=1 is known to give an estimate of the average distance between a random phase in the glassy phase to a nearby gas region, i.e., an estimate of the average domain size [15]. However, in our sample, the oscillations are rather small, impeding an accurate estimation of the domain size.

We also report the static structure factor S(q) obtained as the Fourier transform of the pair correlation function in Figure 2b. S(q) exhibits a peak at low *q* values, which denotes the building of large domains in the system. In agreement with previous works on atomic systems [7,16,17], the amplitudes of the peaks grow with the aging time, and their positions shift towards lower *q* values. The low-q peak hides the one of the dense phase at $q_{max}\approx 2\pi /r_{nb}$, $r_{nb}$ being the mean distance between monomers (see the inset of Figure 2b).

We point out that qualitatively equivalent behavior can be observed also for different quench temperatures, both close to the glass transition and for deeper quenching.

An effective way to measure the typical pores size resorts to the so-called chord length distribution (CLD) [15]. A chord is defined as the segment between two consecutive intersections of the gas–glass interfaces with a virtual line drawn through the system. We employed a set of parallel randomly drawn lines along the three axes and measured the lengths *l* of the segments belonging to the gas phase, which provides more accurate results than the dense phase at short times [7]. It is known that the distribution of the chord lengths P(l,t) is closely related to the distribution of free volume in the material [15]. To estimate the average sizes of the pores, we focus on the first moment of the distribution:

(3) $$L\left(t\right)=\int_{0}^{\infty }dlP\left(l,t\right)l$$

We studied the time evolution of the average pores size for various state points. An example of L(t) is reported in Figure 2c. For short times, we observe slow growth, which could be related to the presence of bonds between particles. Indeed, at the beginning of the coarsening process, the energy required for the creation of interfaces is presumably increased by the constraints imposed by connectivity between atoms. This effect recalls the frozen period in the viscoelastic phase separation [18]: for short times after the quench, no macroscopic domains are formed. Since, in the present work, the temperatures considered are below the predicted glass transition, [19], we cannot exclude that viscoelastic effects could play a role.

At later times, the coarsening process takes over. The domain growth follows a power-law in time with an exponent that is approximately 1/2. As reported in previous works on atomic systems [7,20], this can be seen as effective power-law growth interpolating between two regimes, $t^{1/3}$ for short times and $t^{1}$ for long times. These power laws are the theoretical prediction for the spinodal decomposition: the $t^{1/3}$ regime corresponds to a surface-tension-driven coarsening, and the $t^{1}$ regime corresponds to the hydrodynamic regime. Yet, these regimes do not occur in the state points studied in our work. The absence of the hydrodynamic regime is, however, expected, due to the high viscosity at low temperature, which suppresses hydrodynamic effects. As the $t^{1/2}$ behavior is common among a variety of systems that undergo gas–liquid phase separation under deep quenching, from single-component atomic systems to colloidal suspension [17,21,22,23], it is natural to deem a universal physical mechanism behind this power-law coarsening [5].

To go beyond in this analysis, we explored different state points of the system by changing systematically the chain length *M*, the density ρ and the quench temperature *T*. In Table 1, we report for each set of state parameters the exponent of the intermediate time power-law regime, with the corresponding time window in which the exponent is determined. We note that in the majority of the systems, the domain growth complies with the $t^{1/2}$ law within the exponent incertitude. Deviations are apparent for the lowest quench temperature, T=0.015: the coarsening process was set up at later times and characterized by higher time dependence as compared to the higher temperatures. The origin of this behavior is not clear; as a possible explanation, we can think of a more homogeneous growth mechanism controlled by weaker thermal fluctuations. Further, increasing the molecular weight appears to slow down the coarsening process, as one would expect assuming that connectivity makes rearrangements more complex.

At longer times, the growth of L(t) is slowed down, changing to a logarithmic time dependence in agreement with what reported in atomistic simulations [7]. It would be interesting in future works to explore longer chains to better characterize this evolution.

### 3.2. Mechanical Response

In this section, we characterize the mechanical responses of the porous structures under external deformation, in both the elastic and plastic regimes. Deformation was exerted using the athermal quasi-static (AQS) protocol [24], i.e., by an alternating homogeneous deformation step (uniaxial or shear), with energy minimization (using the conjugate gradient algorithm) to maintain the system at mechanical equilibrium. Since the system was allowed to relax to a new energy minimum before a new strain increment was applied, the AQS protocol corresponds to the zero-shear rate limit.

**Figure 3:**
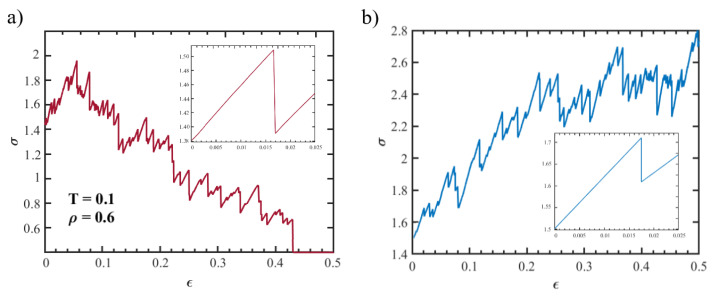
Stress–strain curves for a system at T=0.1, ρ=0.6 and age t=10 in the case of compressive (panel **a**) and tensile (panel **b**) loading. The insets are magnifications of the elastic regions at a small rate of deformation.

Studies on the elastic properties of porous glassy systems have been performed before, using different methods and in different frameworks [8,9,25]. We adopted a "mesoscale approach", in which the elastic properties of the system are obtained from the stress–strain curves of the system restricted to deformations in the genuine elastic regime (approximately below 1%). The deformations were carried out under tensile, compressive and shear loading. In Figure 3, we show representative curves for tensile (panel a) and compressive tests (panel b). The Young’s modulus *E* is estimated as the slope of the curves in the very elastic regime where linear behavior is detected (insets of Figure 3). To reduce the incertitude, assuming system isotropy, *E* is averaged along three deformation directions *x*, *y* and *z*. Further, simple shear tests were also performed to evaluate the shear modulus *G*, for which the average was found in the xy, xz and yz directions.

**Figure 4:**
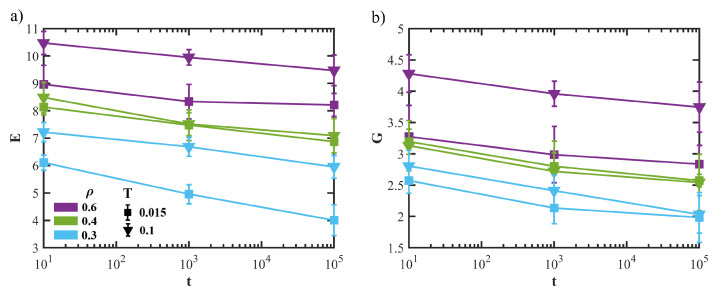
(**a**) Evolution of the elastic constant, Young’s modulus *E* (panel **a**) and shear modulus *G* (panel **b**), with the system’s age for different state parameters (density and quench temperature).

In Figure 4 we show the dependence on the aging time of the Young’s modulus *E* (panel a) and shear modulus *G* (panel b) for states with different densities and quench temperatures. First, we note that both the moduli decrease with aging: the decrease is apparently linear in a semi-log plot, which could signal logarithmic aging. We expect the elastic properties of the system to be controlled by two competing effects: the glassy aging of the dense phase, which leads to the stiffening of the material, and the coarsening process, which promotes a softer overall structure. Then, the reported decreases in *E* and *G* suggest the latter effect to be dominant. Further, it is worth mentioning that in the dynamically arrested configurations, which serve as starting point for mechanical tests, the coarsening process could be reactivated by the external mechanical driving. Yet, we expect this effect to be negligible in the elastic regime, in agreement with previous results on atomic systems [9].

For larger deformations, the elastic response breaks: stress–strain curves appear intermittent, showing alternating elastic loading and sudden stress drops (see Figure 3). The occurring of these stress drops is commonly observed in amorphous systems under deformation and is known to correspond to avalanches of plastic events. It is interesting to examine the distribution of the stress drop magnitude Δσ. Indeed, previous works on amorphous systems reported power-law distributions for stress drops, $P\left(\Delta \sigma \right)∼\Delta \sigma^{-\tau }$ with characteristic exponents in the range τ∼1.2−1.5 [26]. In particular, experimental observations of brittle fractures in amorphous systems found τ∼1.4 [27,28]. Numerical simulations also reported values that ranged from τ∼1.20 for 2D systems to τ∼1.43 for 3D systems [26].

**Figure 5:**
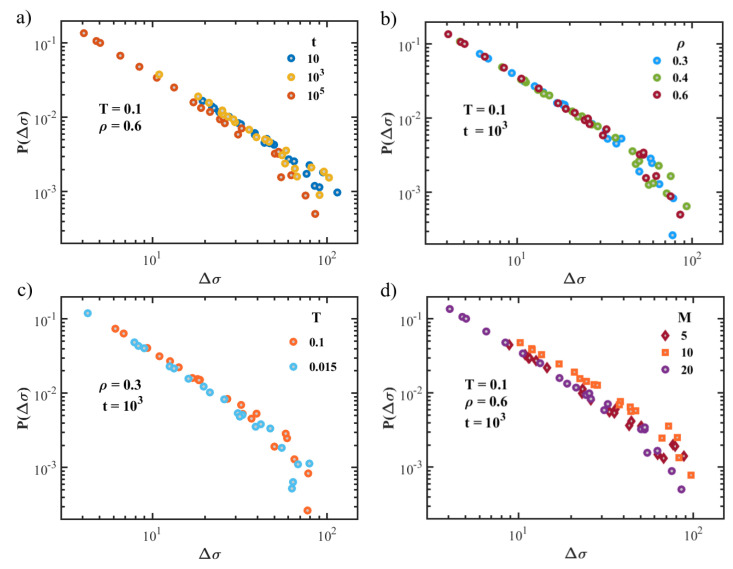
Distribution P(Δσ) of the stress drops in the plastic regime of mechanical response, exploring different combinations of state parameters. Specifically, while keeping fixed all the other parameters, we changed the age (panel **a**), the density (panel **b**), the quench temperature (panel **c**) and the chain length (panel **d**).

In Figure 5, representative stress-drop distributions P(Δσ) are shown for states with different aging times (panel a) densities (panel b) and quench temperatures (panel c). The distribution shows power-law behavior, with an exponential cut due to the finite size of the system. Noticeably, we estimated an exponent τ∼1.45, which seems not to depend on the state parameters and which agrees with the one reported in previous numerical studies of 3D systems [26]. In Figure 5d, we also plotted the distribution of stress drops for systems with different chain lengths M=5,10,20. We note that even for shorter chains, the exponent seems not to deviate significantly, being τ∼1.5 for the specific case M=5.

## 4. Conclusions

The structure and mechanical properties of a porous polymer material were thoroughly investigated using molecular dynamics simulations. Via arrested phase separation, we generated systems consisting of interpenetrating bicontinuos gas–solid phases—the latter being characterized by an amorphous structure. We investigated the time evolution of the coarsening process, which exhibits, at high quench temperatures (but below the glass transition one), the power-law behavior observed in atomic systems, that could be related to the viscoelastic nature of the mechanism.

Then, we analyzed the elastic and plastic responses of the system to external deformation in the athermal quasi-static limit for different densities, quench temperatures and aging times. The analysis of elastic constants revealed the predominance of coarsening over the glassy aging, which led to a softer structure (lower bulk and shear moduli) as the material aged. For large deformations, the plastic response is apparent, resulting in an intermittent pattern of the stress–strain curves. We retrieved a stress-drop distribution which follows a power-law behavior with an exponent τ∼1.45, which is rather robust, not depending on state parameters or system age, and which is compatible with previous numerical and experimental results.

This work represents the first analysis of the effects of chain connectivity on the phenomenon of arrested phase separation. It raises interesting questions, such as the influences of bond stiffness, chain architecture and entanglement effects, which could be the focus of future studies.

## Figures and Tables

**Table 1:** Exponent of the power-law regime in domain growth, and the corresponding time interval in which the exponent is determined, for different states obtained while varying chain length *M*, density ρ and quench temperature *T*.

M, ρ, T	Exponent	Time Window
5, 0.3, 0.3	0.5±0.1	[1000–16,000]
10, 0.3, 0.3	0.5±0.1	[500–16,000]
20, 0.3, 0.3	0.50±0.04	[500–16,000]
5, 0.4, 0.1	0.65±0.09	[500–16,000]
10, 0.4, 0.1	0.56±0.06	[500–16,000]
20, 0.4, 0.1	0.50±0.08	[500–16,000]
5, 0.6, 0.015	0.9±0.1	[2000–100,000]
10, 0.6, 0.015	0.8±0.1	[4000–100,000]
20, 0.6, 0.015	0.56±0.02	[2000–100,000]

## Data Availability

The data presented in this study are available on request from the corresponding author.
